# Preparation and Physicochemical Characteristics of Thermo-Responsive Emamectin BenzoateMicrocapsules

**DOI:** 10.3390/polym9090418

**Published:** 2017-09-05

**Authors:** Yue Shen, Yan Wang, Xiang Zhao, Changjiao Sun, Bo Cui, Fei Gao, Zhanghua Zeng, Haixin Cui

**Affiliations:** Institute of Environment and Sustainable Development in Agriculture, Chinese Academy of Agricultural Sciences, Beijing 100081, China; shenyue@caas.cn (Y.S.); wangyan03@caas.cn (Y.W.); zhaoxiang@caas.cn (X.Z.); sunchangjiao@caas.cn (C.S.); cuibo@caas.cn (B.C.); gaofei@caas.cn (F.G.); zengzhanghua@caas.cn (Z.Z.)

**Keywords:** polydopamine, poly(*N*-isopropylacrylamide), thermo-responsive, microcapsule, controlled-release

## Abstract

Thermo-responsive release emamectin benzoate microcapsules were successfully prepared with a polydopamine (PDA)-*g*-poly(*N*-isopropylacrylamide) (PNIPAm) multifunctional layer. Preparation of emamectin benzoate microcapsules was first studied by emulsion interfacial-polymerization using PDA as a wall material. Then the amino-terminated PNIPAm was grafted on the PDA layer by its amino group in aqueous solution. Physicochemical characterization of microcapsules was obtained by scanning electron microscopy (SEM), transmission electron microscopy (TEM), Fourier transform infrared spectroscopy (FTIR), X-ray photoelectron spectroscopy (XPS), and dynamic light scattering (DLS). Kinetic study of emamectin benzoate release showed that the microcapsules exhibit sustained- and controlled-release properties. The multifunctional layer can release emamectin benzoate easily when the temperature was below the lower critical solution temperature (LCST). In contrast, when the temperature increased above the LCST, the release rate was reduced. The results indicated that these microcapsules with excellent thermo-sensitivity would be promising in the research field of pesticide microcapsules.

## 1. Introduction

In the past few decades, stimuli-responsive release technology has obtained a growing scientific interest worldwide and attracted considerable attention [1]. Stimuli-responsive polymers are capable of adapting to their surrounding environments, regulating ions and molecules transport, changing the adhesion ability of diverse species, or converting chemical and biochemical signals into thermal, electrical, and mechanical signals [2,3,4,5]. With the development of stimuli-responsive release technology, an increasing number of products which issue a response with precise quantities at precise times have been marketed [2].

Conventional applications of agrochemicals contain concentrations far in excess of that required, and leads to toxicity problems and environmental pollution [6]. The substitution of stimuli-responsive release systems for conventional agrochemical formulations helps to avoid the use of excess amounts of active ingredients, and offers ecologic and economic advantages [7]. Among these technical solutions, controlled-release pesticides formulation is satisfying for obtaining the most effective use of pesticide and reducing environmental pollution [8,9]. These formulations are classified into four different forms: microspheres, microcapsules, matrices with physical-trapped pesticides, and polymers with covalently bound pesticides [1,10]. In these systems, microencapsulation is a generalized technology in which polylactic acid, polyurethane, and silica-alginate have been widely employed as the carriers of pesticides [11,12,13]. Many achievements have been obtained in the research of pesticide encapsulation for controlled release [14,15,16].

Polydopamine (PDA) is secreted from mussels as a mimic of the specialized adhesive mytilusedulis foot protein-5 [17]. It can modify the surface of various materials including metal oxides, semiconductors, noble metals, ceramics, and synthetic polymers [18]. Moreover, PDA coating has excellent biocompatibility and low toxicity, making it useful for cells adhesion and encapsulation [19]. PDA capsules can be prepared by the spontaneous oxidative polymerization of dopamine on liquid or solid templates [20]. The capsules exhibit negligible toxicity and have a wide application prospect for drug delivery [21,22,23]. Notably, several reports have shown that PDA capsules are capable of controlling the pesticide release and adjusting the adhesion properties on leaves, prolonging foliar pesticide retention, minimizing its volatilization, and improving itsresidence time on crop surfaces. Thisconceptual strategyprovides a novel pesticide encapsulation method [24,25].

Poly(*N*-isopropylacrylamide) (PNIPAm) is a kind of thermo-responsive polymer [26]. The lower critical solution temperature (LCST) is lower and about 32 °C in aqueous solution [27]. Meanwhile, the network structure of PNIPAm demonstrates that unique thermal volume transitions near its LCST [28]. Because of the specific swelling properties in response to temperature, PNIPAm and its copolymers can be used for controlled release [29,30].

Emamectin benzoate belongs to the avermectin series of natural products [31]. It is macrocyclic lactone insecticide and contains avermectin B1a benzoate (at least 90%) and B1b benzoate salts (at most 10%). Compared with traditional insecticide, emamectin benzoate exhibits a better insecticidal effect. Nevertheless, due to the sensitivity to UV light as well as strongly alkaline and acidic conditions, the biological activity of emamectin benzoate is limited in application [32]. Thus, the use of microcapsules has been widely applied. Microcapsules could protect emamectin benzoate against photo- and thermal degradation effectively. Particularly, PDA microcapsules are capable of controlling the emamectin benzoate release and adjusting the adhesion properties on leaves.

In this research, a simple approach for the preparation of mussel-inspired thermo-responsive emamectin benzoate microcapsules was investigated. The preparation of microcapsules was first studied by emulsion interfacial-polymerization using PDA as a wall material. Then the amino-terminated PNIPAm was grafted on the PDA layer by its amino group in aqueous solution. Finally, the microscopic and surface properties, chemical changes, temperature controlled-release, and adhesive properties of the generated microcapsules were fully characterized.

## 2. Materials and Methods

### 2.1. Materials

Emamectin benzoate (72%) was purchased from Hebei Veyong Bio-Chemical Co., Ltd., China. Dopamine hydrochloride and hexadecyltrimethylammonium chloride (CTAC) were supplied by J&K Scientific Ltd., Beijing, China. Tris-(hydroxymethyl) aminomethane, hydrochloric acid, ethanol, and *n*-butyl alcohol were purchased from Beijing Chemical Reagents Company, China. Poly(*N*-isopropylacrylamide) with an amino end group (molecular weight = 2500) was supplied by Sigma-Aldrich, Inc., St. Louis, MO, USA. Chromatography grade of methanol and acetonitrile were purchased from Fisher (Hampton, NH, USA) and used for high-performance liquid chromatography (HPLC) (Aglient 1260, Santa Clara, CA, USA). All experiments used Milli-Q water (15 MΩ·cm, total organic carbon ≤4 ppb).

### 2.2. Preparation of PDA-Coated Emamectin Benzoate Microcapsules

Briefly, 1.5 g CTAC was dissolved in 100 mL Tris-HCl (pH = 8.5) buffer solution in a flask to obtain the water solution of surfactant. Subsequently, 0.13 g emamectin benzoate was dissolved in 10 mL of *n*-butyl alcohol. In this research, *n*-butyl alcohol containing emamectin benzoate is hereafter defined as the internal organic phase. The solution of 0.13 g emamectin benzoate and 10 mL *n*-butyl alcohol was emulsified in the prepared aqueous solution of surfactant at 1500 rpm for 30 min to form an oil/water (O/W) emulsion. Then, 0.1 g of dopamine was added into the solution. The whole system was stirred at room temperature for one day. The solid microcapsules were obtained by centrifugation and thoroughly washed with water and ethanol at least three times. Then the products were dried to constant weight at 45 °C.

### 2.3. Synthesis of PNIPAm-g-PDA Emamectin Benzoate Microcapsules

PDA-coated emamectin benzoate microcapsules were added into a system in which PNIPAm with an amino end group was dissolved in a 15 mmol·L^−1^ Tris-HCl buffer solution. The grafting reaction occurred at 60 °C for 3 h. When the reaction was completed, the solution was further kept on the shaker for 16 h at room temperature. The modified microcapsules were washed with ultrapure water, and dried at 45 °C to constant weight, yielding PNIPAm-*g*-PDA microcapsules.

### 2.4. Microcapsules Characteristics

#### 2.4.1. Morphology and Structure of Microcapsules

The morphology of emamectin benzoate microcapsules was characterized by scanning electron microscopy (SEM, Hitachi SU8010, Hitachi Ltd., Chiyoda-ku, Japan) with an accelerating voltage of 5 kV. The prepared microcapsules were dispersed in water evenly. The samples were dropped onto cleaned silicon slice surface and dried at room temperature. Then the silicon slice was coated with a thin layer of platinum for 30 s for SEM measurement.

The structure of emamectin benzoate microcapsules was characterized by transmission electron microscopy (TEM, Hitachi HT7700, Hitachi Ltd., Chiyoda-ku, Japan) with an accelerating voltage of 80 kV. The prepared microcapsules were dispersed in water evenly. Then the solution were cast onto a carbon-coated copper grid and evaporated under vacuum at room temperature for TEM measurement.

#### 2.4.2. Chemical Changes on the Microcapsules with Different Shell Material

Fourier transform infrared spectroscopy (FTIR) experiments were conducted with a Nicolet IS10 spectrometer (Thermo Fisher Scientific, Waltham, MA, USA). X-ray photoelectron spectroscopy (XPS) was performed using an Axis Ultra spectrometer (Thermo escalab 250XI, Thermo Fisher Scientific, Waltham, MA, USA), which was equipped with a monochromatic Al Ka X-ray source of 150 W (*hv* = 1486.6 eV). The assumed C 1s peak was at 284.8 eV. The charge shift of the spectra was corrected by the C 1s peak.

#### 2.4.3. Mean Diameter and Zeta Potential of Microcapsules

The mean diameter of the emamectin benzoate microcapsules was measured by the dynamic light scattering (DLS) technique with a ZetasizerNano ZS90 (Malvern Instruments, Malvern, UK) in water solution. The sizes of the microcapsules were tested at different temperatures. The swelling ration (SR) of the PNIPAm-*g*-PDA microcapsules was calculated according to the mean diameters of microcapsules at 25 or 37 °C (*D*_h25_ or *D*_h37_) on the basis of the equation: SR = *V*_swollen_/*V*_shrunken_ = *D*_h37_/*D*_h25_. *V*_swollen_ and *V*_shrunken_ representthe volumes of microcapsule at 37 and 25 °C, respectively [25]. The zeta potential of the emamectin benzoate microcapsules was measured by the same instrument.

#### 2.4.4. Determination of Amount of Emamectin Benzoate Encapsulated in PDA

To determine the amount of emamectin benzoate encapsulated in PDA, the percentages of the emamectin benzoate loading and encapsulated were measured as follows: a given mass of PDA-coated microcapsules were blended with 50 mL of methanol solution for 24 h with magnetic stirring. The concentration of emamectin benzoate was examined by HPLC (Aglient 1260, Santa Clara, CA USA) with a C18 column (5 μm, 4.6 mm × 150 mm, Aglient, Santa Clara, CA, USA) and a 245-nm UV detector. The organic solution was diluted and filtered through a 0.2-mm filter prior to HPLC analysis. Then the percentages of emamectin benzoate loading and encapsulation were evaluated using the following equations: emamectin benzoate loading = (mass of emamectin benzoate loaded in PDA/mass of microcapsules) × 100%, emamectin benzoate encapsulation = (mass of emamectin benzoate loaded in PDA/mass of emamectin benzoate) × 100%.

#### 2.4.5. Controlled Release of PDA-Coated Emamectin Benzoate Microcapsules

The release behavior from different types of PDA-coated emamectin benzoate microcapsules was evaluated as follows: 0.02 g of microcapsules was added into dialysis bags for which the molecular weight cut-off (MWCO) was 8000–14,000. Next, the bags were put into conical flasks with 200 mL of methanol/H_2_O (1:1, *v*/*v*) solution and shaken at the temperatures of 25 and 37 °C. Then the mixtures were placed in incubators and shaken at a speed of 100 rpm constantly. The cumulative release rate of emamectin benzoate from the PDA-coated microcapsules was evaluated by the concentration of emamectin benzoate dissolved in the system at different times. 

#### 2.4.6. Studies of the Adhesion Properties of PDA-Coated Emamectin Benzoate Microcapsules

Two hydrophobic silicon wafers were immersed into the aqueous suspension of emamectin benzoate microcapsules solution for 30 min and dried in air. One wafer was washed with deionized water for 30 min, with the other undergoing no further treatment. Both were dried under vacuumat 45 °C for one day.

## 3. Results and Discussion

### 3.1. Preparation of PNIPAm-g-PDA Emamectin Benzoate Microcapsules

**Figure 1:**
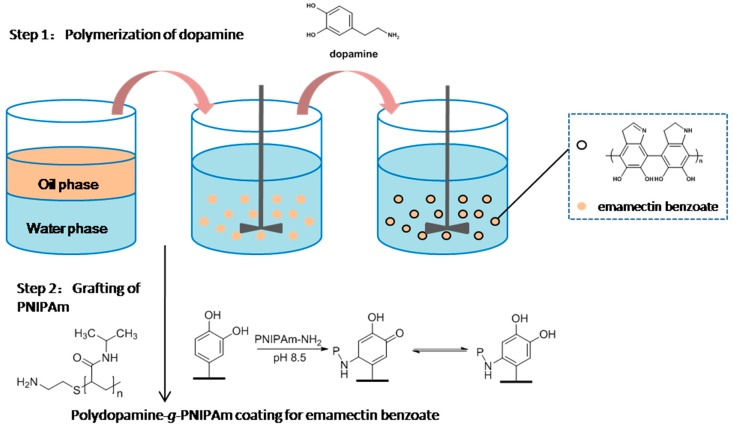
Schematic illustration of the preparation of thermo-responsive microcapsules.

Figure 1 displays the preparation process of thermo-responsive PNIPAm-*g*-PDA emamectin benzoate microcapsules. The strategy involves three key steps. First of all, emamectin benzoate and *n*-butyl alcohol composed the oil droplets. CTAC was utilized to stabilize the solution as one kind of surfactant. Then self-polymerization of dopamine through pH-induced oxidation occurred on the water-oil interface to form the outer shell of microcapsules at room temperature. The color of PDA deepened as the reaction time prolonged because of the strong UV-visible absorption [17]. 

When dopamine is used for surface modification, the surface hydrophilicity can be promoted effectively [17]. Meanwhile, molecules and polymers which contain free amine groups are easily covalent immobilized on dopamine [33]. Thus, the active PDA layer obtained before was used as an intermediate to graft PNIPAmin order to achieve thermo-responsive microcapsules. The PDA-coated emamectin benzoate microcapsules were dipped into amino-terminated PNIPAm aqueous solution, followed by the spontaneous grafting reaction. During the process, the color of the product changed from dark brown to gray.

### 3.2. Characterization of PNIPAm-g-PDA Emamectin Benzoate Microcapsules

**Figure 2:**
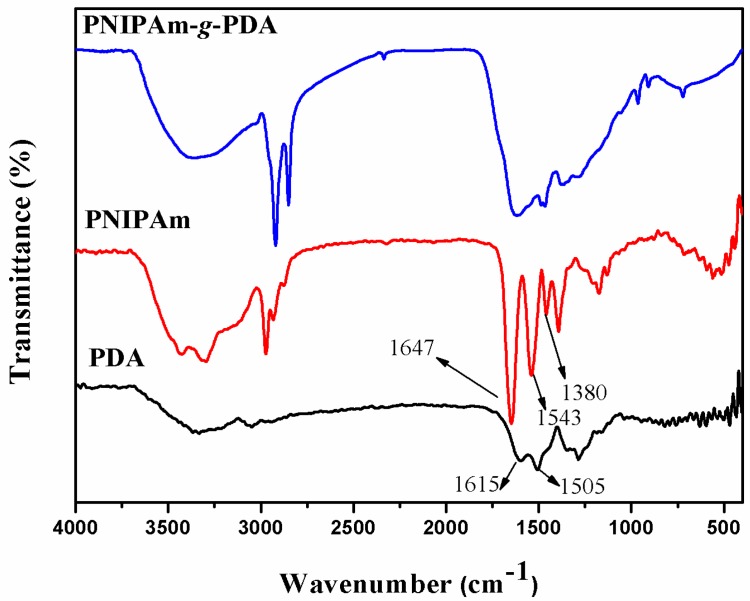
FTIR spectra of PDA, PNIPAm and PNIPAm-*g*-PDA.

Figure 2 depicts the FTIR spectra of PNIPAm, PDA, and a hollow grafted microcapsule without emamectin benzoate. From the spectra, PDA presents absorption peaks at 1505, 1615, and 3150–3400 cm^−1^ from the indole/indoline structures and N−H/O−H bonds, as expected [34]. From the PNIPAm spectrum, the peak at 1380 cm^−1^ is ascribed to C−H bonds in the methyl groups of isopropyl. Additionally, the characteristic bands at 1543 and 1647 cm^−1^ are attributed to the amide II band (δ, N−H) and amide I band (ν, C=O) [35]. Moreover, as for the PDA-*g*-PNIPAm spectrum, the PDA and PNIPAm characteristic groups also exist in the results. These results confirmed that the grafting reaction occurred between PDA and PNIPAm, leading to the formation of PNIPAm-*g*-PDA successfully.

**Figure 3:**
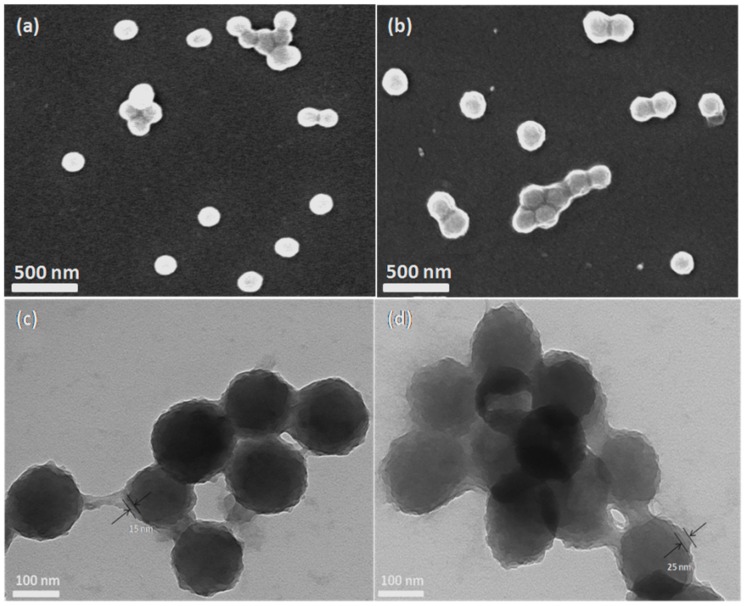
SEM and TEM images of the samples: (**a**,**c**) PDA-coated microcapsules and (**b**,**d**) PNIPAm-*g*-PDA microcapsules.

The SEM and TEM images of PDA-coated microcapsules and PNIPAm-*g*-PDA microcapsules are given in Figure 3. The images show that the PDA-coated microcapsules adhered to each other and showed smooth surfaces (Figure 3a,c). The mean size of microcapsules was about 130 nm. The boundary of encapsulation was clear and the PDA layer was successfully coated on the water-oil interface. The average thickness of the layers was about 15 nm. After the grafting reaction of PNIPAm, thermo-responsive microcapsules were formed. The layer thickness was increased to about 25 nm accordingly (Figure 3b,d).

**Figure 4:**
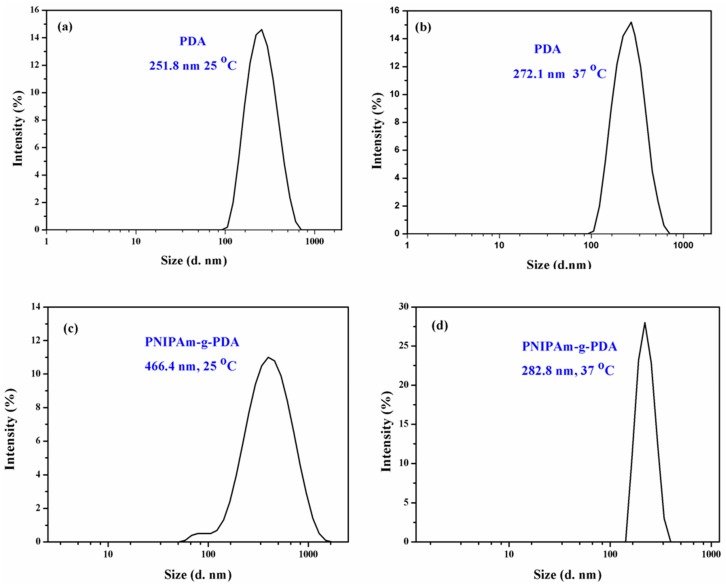
The size distribution of (**a**,**b**) PDA-coated and (**c**,**d**) PNIPAm-*g*-PDA microcapsules under different conditions.

The size distribution of PDA-coated and PNIPAm-*g*-PDA microcapsules was determined. Figure 4a–d display the hydrodynamic diameter (*D*_h_) of the microcapsules at 25 or 37 °C. The diameters of the PDA-coated microcapsules at 25 and 37 °C were about 252 and 272 nm, respectively. The diameter of the PNIPAm-*g*-PDA microcapsule at 25 °C was about 466 nm. However, the diameter of the PNIPAm-*g*-PDA microcapsule was reduced to about 283 nm at 37 °C because of the collapse of the PNIPAm chains. Calculated from the equation, the SR of the PNIPAm-*g*-PDA microcapsules was 1.65. These results demonstrate that the PNIPAm-*g*-PDA microcapsules exhibited good thermo-responsive capabilities. When the temperature was above the LCST, the size of the PNIPAm-*g*-PDA microcapsule was smaller than that below the LCST. This difference can also be attributed to the improvement of the hydrophilicity of the microcapsule [17]. In comparison with the zeta potential of the PDA microcapsule without PNIPAm (−18.7 mV), the PNIPAm-*g*-PDA microcapsule (11.7 mV) changed markedly. The results also showed that PNIPAm was successfully grafted to the PDA layer.

**Figure 5:**
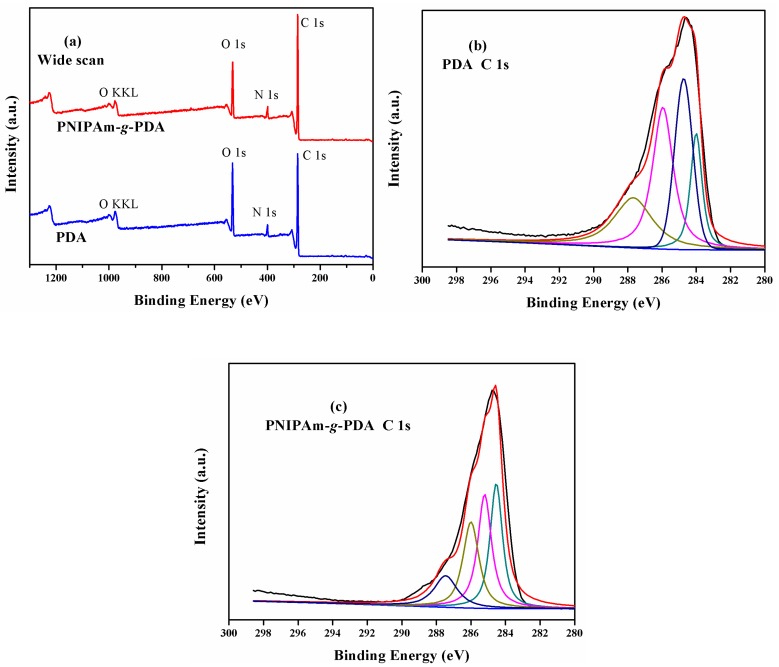
Wide-scan XPS spectra (**a**) and C 1s (**b**,**c**) high-resolution XPS element spectra of PDA-coated microcapsules and PNIPAm-*g*-PDA microcapsules.

XPS was employed to study the surface composition of emamectin benzoate microcapsules. The different elements binding states which formed the surface layer were analyzed by the high-resolution element spectra. As can be seen in Figure 5a, after polymerizing dopamine to form the outer shell, the unmodified microcapsule contains elements carbon, oxygen, and nitrogen, which are expected elements of dopamine. At the same time, the element contents of C, O, and N were 73.94%, 14.94%, and 6.12% in PDA, respectively. The C 1s spectrum of the unmodified PDA microcapsule shows an intensive shoulder at about 285 eV (Figure 5b). The component peaks at 285.95 and 284.01 eV resulted from the phenolic C–OH groups and the cyclic amine bonds (C–NH–C/C–N=C), respectively [29]. Additionally, the component peak at 287.68 eV can be clearly observed. The origin of the peak was a certain amount of nitrogen in amide groups. Otherwise, the component peak Ph at 284.01 eV shows the carbon atoms of the phenyl rings of PDA.

For the grafted polymer, the element contents of C, O, and N turned to 78.66%, 15.82%, and 5.52%, respectively. Additional amide groups were introduced by the grafting of PNIPAm. Thus, the relative element content [N]:[C] on the microcapsule surface increased slightly. In the high-resolution C 1s spectrum, the intensity of the component peak at 285.21 eV was increased by the amine-sided carbon atoms of the amide groups of PNIPAm (Figure 5c). The corresponding carbonyl atoms contributed with the same intensity to the component peak at 286.00 eV. These results demonstrate that PNIPAm was successfully grafted on the PDA surface.

### 3.3. Controlled Release Kinetics

**Figure 6:**
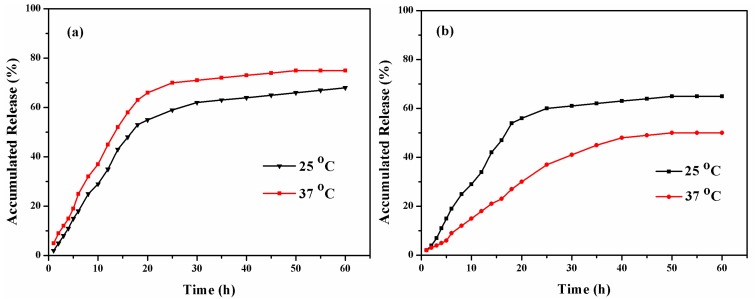
Emamectin benzoate release behaviors from the PDA microcapsule in the release system at various temperatures: (**a**) PDA-coated microcapsules and (**b**) PNIPAm-*g*-PDA microcapsules.

The emamectin benzoate loading capacity and the encapsulation efficiency in the resulting microcapsules calculated from the abovementioned equations was 49% and 58% (*w*/*w*), respectively. The emamectin benzoate release behaviors from PDA-coated and PNIPAm-*g*-PDA microcapsules were investigated at different temperatures (25 and 37 °C) in a methanol/H_2_O (1:1, *v*/*v*) solution stirred at 100 rpm. Figure 6a shows that the release rate of the PDA-coated microcapsules increased with the increase of temperature. The accumulative release rate of emamectin benzoate from the PDA-coated microcapsules was 75 wt % (37 °C) and 68 wt % (25 °C) within 60 h. The results show that the PDA layer has the ability to control the release behavior of entrapped emamectin benzoate. It is worth noting that the accumulative release rate of emamectin benzoate from the prepared samples at high temperatures is faster than that at low temperatures. The reason for this is that molecule diffusion through the PDA shell intensifies at higher temperatures.

However, for the PNIPAm-*g*-PDA microcapsules, the release rate appeared faster at temperatures below the LCST. The achievable maximum release of emamectin benzoate was significantly lower at 37 °C than that at 25 °C (Figure 6b). The accumulative release rate of emamectin benzoate decreased with increasing temperatures. This phenomenon was ascribed to the grafted thermo-sensitive PNIPAm. When the temperature (25 °C) was below the LCST, the PNIPAm polymer swelled and the structure became looser. Thus, the PNIPAm-*g*-PDA microcapsules release performance was more effective. When the temperature (37 °C) increased above the LCST of PNIPAm, the polymer chains rapidly collapsed to prevent the release of emamectin benzoate. In conclusion, the thermo-sensitive PNIPAm polymer played an important role as a nanoreactor that could be opened or closed in order to control emamectin benzoate release.

### 3.4. Adhesion Properties of Emamectin Benzoate Microcapsules

**Figure 7:**
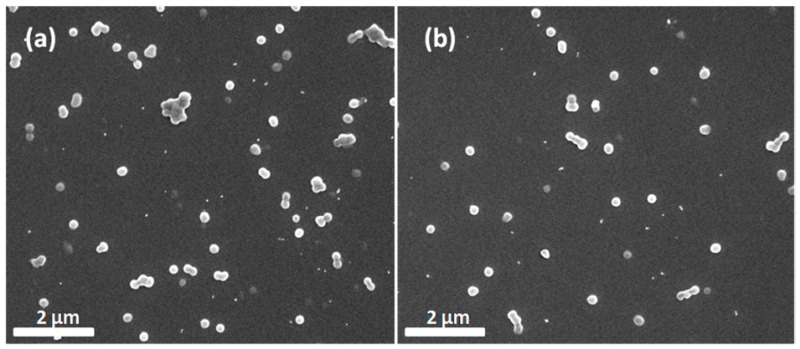
SEM images of hydrophilic silicon slices: (**a**) PDA-coated microcapsules and (**b**) PDA-coated microcapsules after washing.

To further prove that PDA-coated microcapsules have better adhesion behavior, the samples were sprayed on smooth silicon slices. The amount of samples remaining on the silicon surface was observed to evaluate the adhesion properties of microcapsules. SEM images (Figure 7a,b) show that the amount of PDA-coated microcapsules barely changed on the silicon slices before (83) and after water washing (48). The results demonstrate that PDA-coated microcapsules have excellent adhesion properties. This may be attributed to a great quantity of catechol groups in the PDA structure, which enhance the adhesion performance on silicon slices [36].

## 4. Conclusions

PNIPAm-*g*-PDA stimuli-responsive release microcapsules were successfully prepared through a facile and generalized approach. The multifunctional layer formed by the PDA inner film and thermo-sensitive PNIPAm outer corona exhibited stimulative responsibility for encapsulated emamectin benzoate depending on the temperature. The PNIPAm-*g*-PDA layers could release emamectin benzoate easily when the temperature was below the LCST. In contrast, when the temperature increased above the LCST, the release rate was reduced. The results indicated that the PNIPAm-*g*-PDA controlled-release microcapsules with excellent thermo-sensitivity could be expected to be promising materials in the research and development of pesticide microcapsules.
